# Lateral Symmetry of Synergies in Lower Limb Muscles of Acute Post-stroke Patients After Robotic Intervention

**DOI:** 10.3389/fnins.2018.00276

**Published:** 2018-04-25

**Authors:** Chun Kwang Tan, Hideki Kadone, Hiroki Watanabe, Aiki Marushima, Masashi Yamazaki, Yoshiyuki Sankai, Kenji Suzuki

**Affiliations:** ^1^Artificial Intelligence Laboratory, University of Tsukuba, Tsukuba, Japan; ^2^Center for Innovative Medicine and Engineering, University of Tsukuba Hospital, Tsukuba, Japan; ^3^Department of Neurosurgery, Faculty of Medicine, University of Tsukuba, Tsukuba, Japan; ^4^Department of Orthopaedic Surgery, Faculty of Medicine, University of Tsukuba, Tsukuba, Japan; ^5^Center for Cybernics Research, University of Tsukuba, Tsukuba, Japan; ^6^Faculty of Engineering, University of Tsukuba, Tsukuba, Japan

**Keywords:** muscle synergies, gait symmetry, stroke rehabilitation, robotic intervention, hybrid assistive limb

## Abstract

Gait disturbance is commonly associated with stroke, which is a serious neurological disease. With current technology, various exoskeletons have been developed to provide therapy, leading to many studies evaluating the use of such exoskeletons as an intervention tool. Although these studies report improvements in patients who had undergone robotic intervention, they are usually reported with clinical assessment, which are unable to characterize how muscle activations change in patients after robotic intervention. We believe that muscle activations can provide an objective view on gait performance of patients. To quantify improvement of lateral symmetry before and after robotic intervention, muscle synergy analysis with Non-Negative Matrix Factorization was used to evaluate patients' EMG data. Eight stroke patients in their acute phase were evaluated before and after a course of robotic intervention with the Hybrid Assistive Limb (HAL), lasting over 3 weeks. We found a significant increase in similarity between lateral synergies of patients after robotic intervention. This is associated with significant improvements in gait measures like walking speed, step cadence, stance duration percentage of gait cycle. Clinical assessments [Functional Independence Measure-Locomotion (FIM-Locomotion), FIM-Motor (General), and Fugl-Meyer Assessment-Lower Extremity (FMA-LE)] showed significant improvements as well. Our study shows that muscle synergy analysis can be a good tool to quantify the change in neuromuscular coordination of lateral symmetry during walking in stroke patients.

## 1. Introduction

Stroke is a serious neurological disease commonly associated with gait disturbance (Verma et al., 2012; Esquenazi et al., 2017). This points to a need for asssitive devices that can help in patients' therapy process and personal mobility. In reviews done by Díaz et al. (2011) and Esquenazi et al. (2017), there appears to be an increasing trend in the use of exoskeletons for therapy. This has led to the development of several commercially available exoskeletons for therapy. Some examples of these exoskeletons are the Hybrid Assistive Limb (HAL) (Hayashi et al., 2005), ReWalk (Zeilig et al., 2012), Lokomat (Jezernik et al., 2003), and Lopes (Veneman et al., 2007).

Although improvements to stroke patients' gait can be clinically verified after robotic intervention, the effects of such interventions from the viewpoint of muscle activations are not well studied. Typically, studies involving the use of exoskeletons focus on clinical metrics describing the performance of patients before and after a course of therapy (Aach et al., 2014; Watanabe et al., 2014). Louie and Eng (2016) did a review on various studies that looked into the effects of exoskeletons on therapy and listed methods and results from them. It was also noted that all of the studies listed in their review used mainly only clinical measures to evaluate improvement in patients. Díaz et al. (2011) also concluded that there is a need to develop standardized protocols to obtain reliable assessment data, as clinical measures are currently not sufficient and require many clinical trials in order to be widely accepted and implemented. Although clinical measures are a good indication of the general wellbeing of patients, they are unable to reflect changes in the way muscles are coordinated in a task. We believe having knowledge in how muscle synergies change after therapy would help determine accurately if the patient has regained their mobility.

One measure where clinical measures do not quantify well is gait symmetry. However, such a measure is turning out to be an important measure to evaluate post stroke patients. Verma et al. (2012) did an extensive review on characterization of such asymmetries and found that asymmetries arise on the unaffected side due to compensation and adaptation. They also found that such asymmetries lead to inefficient energy expenditure, falls, abnormal joint loading, joint pain, deformity and pain. Since gait asymmetry is such a serious issue, having tools like muscle synergy analysis (MSA) would allow us to assess patient gait performance accurately, and thus, customize treatments. Further support for measuring gait asymmetries come from Patterson et al. (2010), who analyzed patient gait data up to a mean of 82 months, post stroke. They found that spatial and temporal gait symmetry parameters (stance time, swing time, step length symmetry), actually show a worsening of gait in those patients, whereas parameters like velocity, neurological deficit and lower extremity motor impairment did not reveal any significant worsening of gait. They conclude that gait asymmetry should be given more attention both in clinical situation and research.

Previous studies have proposed that co-activation of muscles, also known as muscle synergies or motor modules, are sufficient to describe various postural or locomotion tasks in humans (Ivanenko et al., 2007; Torres-Oviedo and Ting, 2007). These synergies can be considered strategies that the human body employs to facilitate control of limbs in various tasks. Such a method, known as muscle synergy analysis (MSA), has recently seen interest in the stroke therapy. Studies by Clark et al. (2010), Barroso et al. (2017), and Gizzi et al. (2011) used MSA to assess gait performance of stroke patients. One related work by Routson et al. (2013) employed MSA to quantify walking performance of stroke patients before and after therapy. These studies highlight the importance of having such measures, in addition to clinical measures, in order to predict stroke patient performance and to customize therapies.

In our study, we investigate muscle activation changes with MSA in stroke patients who underwent a course of robotic therapy using the HAL Lower Limb exoskeleton (Hayashi et al., 2005). Muscle synergies are extracted with Non-Negative Matrix Factorization (NNMF) (Gelsy Torres-Oviedo and Ting, 2006) and compared to evaluate changes in muscle activation of stroke patients before and after robotic therapy. This study aims to quantify gait symmetry of post stroke patients with lateral symmetry of muscle synergies on both sides of the body, when they are walking.

## 2. Methods

This study was carried out in accordance with the recommendations of the University Guidelines for Clinical Trials, Institutional Review Board of University of Tsukuba Hospital, with written informed consent from all subjects. All subjects gave written informed consent in accordance with the Declaration of Helsinki. The protocol was approved by the Institutional Review Board of University of Tsukuba Hospital. The University Guidelines for Clinical Trials conforms to the ethical principles of the Declaration of Helsinki.

### 2.1. Participants

Eight post stroke patients in their acute phase after onset participated in the study (Table 1). Among the eight participants, there were four females and four males, aged between 43 and 80 (average: 59.3 ± 12.2) yrs. Four of them had hemiparesis on the left side, and the other four on the right side. Medical diagnosis included Atherothrombotic Cerebral Infarction (ACI), Subcortical Hemorrhage (SH), Brain Stem Infarction (BSI), Lacunar Infarct (LI) and Atherothrombotic Brain Stem Infarction (ABSI). Clinical evaluation of their Functional Ambulation Category (FAC) before starting the robotic intervention ranged from 1 (ambulation under substantial physical assistance) to 3 (independent ambulation under observation). These patients were included in the study 10–18 (average: 13.6 ± 3.4) days after onset.

**Table 1:** Participants characteristics.

**ID**	**Age (years)**	**Gender**	**Diagnosis**	**Affected side**	**FAC before HAL**	**Onset-HAL duration (days)**
S1	67	F	ACI	L	1	10
S2	52	F	SH	R	2	17
S3	71	F	BSI	L	1	11
S4	55	M	LI	L	2	10
S5	55	F	ABSI	L	3	16
S6	43	M	LI	R	2	11
S7	51	F	ACI	R	2	18
S8	80	M	ACI	R	2	16

*Diagnosis was Atherothrombotic Cerebral Infarction (ACI), Subcortical Hemorrhage (SH), Brain Stem Infarction (BSI), Lacunar Infarct (LI) or Atherothrombotic Brain Stem Infarction (ABSI)*.

### 2.2. Robotic intervention

Since all participants were hemiparetic, single leg version of Robot Suit HAL was used. The robot was composed of three rigid structures corresponding to lumbar, thigh and shank, and shoe, of the paretic side, weighing 9 kg in total. These parts were serially connected by joints allowing relative sagittal motion, realizing joint motion of hip, knee and ankle in the sagittal plane. Electric motors were embedded at the hip and knee joints, and controlled according to the bioelectric signals detected by surface electrodes attached on the skin surface of the relevant muscles. In equation, the hip and knee motors were controlled in real time to provide assistive joint torque as *T*_*hip*_ = *G*_*hip, flex*_ * *A*_*hip, flex*_ − *G*_*hip, ext*_ * *A*_*hip, ext*_ and *T*_*knee*_ = *G*_*knee, flex*_ * *A*_*knee, flex*_ − *G*_*knee, ext*_ * *A*_*knee, ext*_, where *A*_*hip, flex*_, *A*_*hip, ext*_, *A*_*knee, flex*_ and *A*_*knee, ext*_ are respectively filtered activation of Illiopsoas (hip flexor), Gluteus maximus (hip extensor), Hamstrings (knee flexor) and Vastus Lateralis (knee extensor) muscles. *G*_*hip, flex*_, *G*_*hip, ext*_, *G*_*knee, flex*_, and *G*_*knee, ext*_ are gain parameters adjusted according to wearer's comfort through the sessions.

Robotic intervention was started within the participants' acute period (Table 1). Intervention sessions were performed three times per week for 3 weeks, and therefore nine times in total, for each patient. An intervention session lasted approximately 60 min, including clinical examination, attaching the robot, 20 min of walking training using the robot including rest when necessary, and detaching the robot. During walking training, the patient walked repetitively in a 25 m course composed of two straight lines and two semicircles, on a flat surface. For safety reasons, a walking device (All-in-One Walking Trainer, Ropox A/S, Naestved, Denmark) with a harness was used to prevent falls, and heart rate and oxygen saturation were monitored time to time.

### 2.3. Data measurement

Gait of the patients was measured during straight walking at a self-selected speed without wearing HAL, 1–3 days before the first HAL session (pre HAL) and after the last HAL session (post HAL). Lower limb muscle activity and foot motion were recorded using a measurement system. All-In-One Walking Trainer (Ropox A/S, Denmark), with a harness, was used during the walking test to prevent falls. The harness was adjusted so that it did not provide any weight support. The patients walked for 6 m several times (Lam et al., 2010), until three consecutive steady steps were obtained. Data that did not fit the criteria of three consecutive steady steps were discarded. Also, the initiation and termination of walking during each 6 meters walking trial were discarded as well.

#### 2.3.1. Electromyography

Skin preparation included wiping down the muscle bellies with alcohol swabs. Twelve wireless, surface EMG electrodes were placed bilaterally over the muscle bellies of: Vastus Medialis (VM), Hamstrings (HAM), Tibialis Anterior (TA), Gastrocnemius (GAS), Adductor Longus (ADD), Gluteus Maximus (Gmax), using a TrignoTM Lab Wireless electromyography (EMG) system (Delsys Inc., Boston, MA, USA). EMG data was sampled at 2,000 Hz.

#### 2.3.2. Motion capture system

Motion tracking of subjects was achieved with a motion capture system (VICON MX System with 16 T20S Cameras, Vicon, Oxford, UK), in synchronization with EMG and sampled at 100 Hz. Sixteen autoreflective markers were placed bilaterally on the anterior superior iliac spine, posterior superior iliac spine, lower lateral 1/3 surface of the thigh, flexion-extension axis of the knee, lower lateral 1/3 surface of shank, lateral malleolus for the ankle, posterior peak of the calcaneus for the heel and the lateral second metatarsal bone of the toe. These marker positions were used for gait phase detection during locomotion.

### 2.4. Data analysis

#### 2.4.1. Preprocessing

From the synchronized tracks of EMG and motion data, three consecutive steady steps starting from a heel strike and ending with a succeeding heel strike were extracted in the middle of 6 m walking for each leg (Right and Left), pre and post HAL, for each of the participants. EMG data was first band-passed with a 4th order, zero-lag Butterworth band-pass filter at 30–400 Hz. The bandpassed EMG was then filtered with a Hampel filter, with the parameters, time window, win = 200 and a threshold of σ = 4 (standard deviations), to remove artifacts. Finally, EMG data was fully rectified and low-passed with a 4th order, zero-lag Butterworth low-pass filter at 6 Hz to obtain the EMG envelope. The EMG envelope is then time-normalized and resampled to 100 times points.

#### 2.4.2. Extraction of EMG based on kinematic data

We segmented the EMG data into windows based on the phases of walking (Stance, Swing, Cycle). Stance is defined as the period starting from a heel strike and ending with toe off. Swing is defined as the period between starting from the toe off to heel strike. The Cycle is defined as starting from a heel strike and ending at the next heel strike.

Segmented data is further divided into sides (Affected and Unaffected). Each muscle vector in each segment was divided by its own standard deviation in that particular segment (e.g., Cycle muscle vectors are divided with the standard deviation of muscle vectors in the Cycle segment). This is based on the “UnitPer” definition (standard deviation per trial) of Banks et al. (2017), who evaluated the effect of different EMG normalization methods with NNMF MSA. This provides a consistent effect size for varying muscle synergies and timing coefficients.

Data segments from 3 consecutive walking cycles were separated and concatenated, based on their phase in the gait cycle and side of the patient, thus obtaining 6 matrices in total (Affected_Stance, Affected_Swing, Affected_Cycle, Unaffected_Stance, Unaffected_Swing, Unaffected_Cycle). Oliveira et al. (2014) noted that for intra-subject comparisons, muscle synergies extracted from concatenated signals yielded higher reconstruction quality, as compared to muscle synergies from averaged signals. This processing method was adopted as we would like extracted synergies to be representative of the subject's muscle activations. Also, we think averaging the EMG signals would mask step-to-step variability of muscle activations in hemeparetic patients.

#### 2.4.3. Muscle synergy extraction with NNMF

NNMF was then used to extract muscle synergies from the concatenated EMG data. This was performed with Matlab's NNMF function, using the multiplicative update algorithm. Parameters for the tolerance for the residual (TolFun) was given as 1*e*−6 and the tolerance for the relative change in elements (TolX) was given as 1*e*−4. The algorithm was repeated 50 times and results with the lowest root mean square residual were taken to be the best. Synergies were allowed to vary per condition.

The choice of number of synergies were determined with the criteria of when the variance-accounted-for (VAF_*total*_) between the reconstructed and original EMG envelope was above 90% and subsequent increase of the number of synergies did not give more than a 5% increase in VAF. We also imposed a local criteria where the reconstruction VAF (VAF_*muscle*_) for each muscle vector was above 75% (Torres-Oviedo and Ting, 2007). The VAF is defined as 100 * (uncentered Pearson correlation coefficient), which requires the total sum of squares to be taken with respect to zero (Gelsy Torres-Oviedo and Ting, 2006). This is given as:

(1) $$\begin{array}{l} VAF=100\cdot \left(\frac{{\left(\overset{m}{\underset{j=1}{\sum }}\overset{n}{\underset{i=1}{\sum }}X_{nm}\cdot Y_{nm}\right)}^{2}}{\left(\overset{m}{\underset{j=1}{\sum }}\overset{n}{\underset{i=1}{\sum }}X_{nm}^{2}\right)\cdot \left(\overset{m}{\underset{j=1}{\sum }}\overset{n}{\underset{i=1}{\sum }}Y_{nm}^{2}\right)}\right) \end{array}$$

where *n* is the number of datapoints for each channel, and *m* is the number of channels. *X*_*nm*_ and *Y*_*nm*_ are the matrices containing the reconstructed and original signal respectively. VAF calculation code is adapted from the “rsqr_uncentered” function in the file “PosturalData_NMFvsPCA_GUI_July2013” given in Neuromechanics Lab (2016).

The mean muscle VAF was calculated for each extracted muscle synergy vector ($\frac{1}{n}\overset{n}{\underset{i=1}{\sum }}{\text{VAF}}_{muscle}^{i}$, where *i* = number of synergies and *n* = number of muscle channels), and were used as a basis in the sorting of muscle synergy vectors. Synergy vectors were sorted according to the mean muscle VAF in descending order (i.e., a synergy with highest mean reconstruction muscle VAF, as compared to other synergies, were placed as the first synergy).

#### 2.4.4. Synergy analysis

Lateral synergy symmetry was determined by comparing the sorted synergies (described in section 2.4.3) from the affected side and unaffected side with the general Pearson correlation coefficient (*r*). Muscle synergy matrices were compared with the corresponding synergy matrix for the other side of the body (e.g., Synergy_*affected*_ with Synergy_*unaffected*_ and so on). Such comparisons were performed for muscle synergies extracted from the concatenated EMG data (section 2.4.2) during different phases of gait. Note that only muscle synergies that belong to the same gait phase were compared (e.g., muscle synergies from the full cycle phase, on the affected side of the body, were compared only with the muscle synergies from the full cycle phase, on the unaffected side of the body). The motivation for this comparison is to provide a single value measure for similarity.

Synergy vector comparison with the scalar dot product (Cheung et al., 2012) was also performed to evaluate the changes in contents of the muscle synergy vectors. Muscle synergy matching was also performed to discover the presence of similar muscle synergy vectors on both sides of the body. Muscle synergies with the highest scalar dot product score are selected, matched and removed from the pool of muscle synergies. This process continues until no more muscle synergies are left to match.

#### 2.4.5. Software

Data extraction was done using scripts on MATLAB 8.4 (Mathworks, Natick, MA, USA). NNMF and the rest of the processing were performed with scripts on MATLAB 9.3 (Mathworks, Natick, MA, USA).

### 2.5. Clinical assessments

Physical therapists also evaluated the patients before and after the course of therapy. The below measures were used to evaluate patient motor functions:

1. Functional Independence Measure-Locomotion (FIM-Locomotion)
2. Functional Independence Measure-Motor (General) [FIM-Motor (General)]
3. Fugl-Meyer Assessment, Lower Extremity (FMA-LE)

For the kinematics, we measured the walking speed, step cadence, absolute lateral difference of step length and the percentage of stance in relation to gait cycle. The absolute lateral difference of step length was derived from the step length variable. The absolute difference in step length between both sides of the body was calculated to account for the differences in compensatory walking strategies employed by the patients. Step length does not take into consideration the compensatory gait patterns hemiparetic patients exhibit. For example, some patients start their swing phase with their affected leg and bring their unaffected leg to their center for stabilization (Verma et al., 2012). This causes the affected step length to be longer than the unaffected side. Similarly, patients who drag their affected leg during walking would have a longer step length on their unaffected side.

### 2.6. Statistical analysis

Normality of the data were tested with the Shapiro-Wilk test, with the significance level set to 5%. Statistical comparison performed with the *T*-test (for normally distributed data) and the Mann-Whitney *U*-test (for non-normal distribution). For each paired dataset, both pairs would have to fulfill the criteria of the Shapiro-Wilk test before the *T*-test was applied. Otherwise, the *U*-Test was used instead. Significance was considered in comparisons with *p* < 0.05.

## 3. Results

### 3.1. Clinical assessments

**Figure 1:**
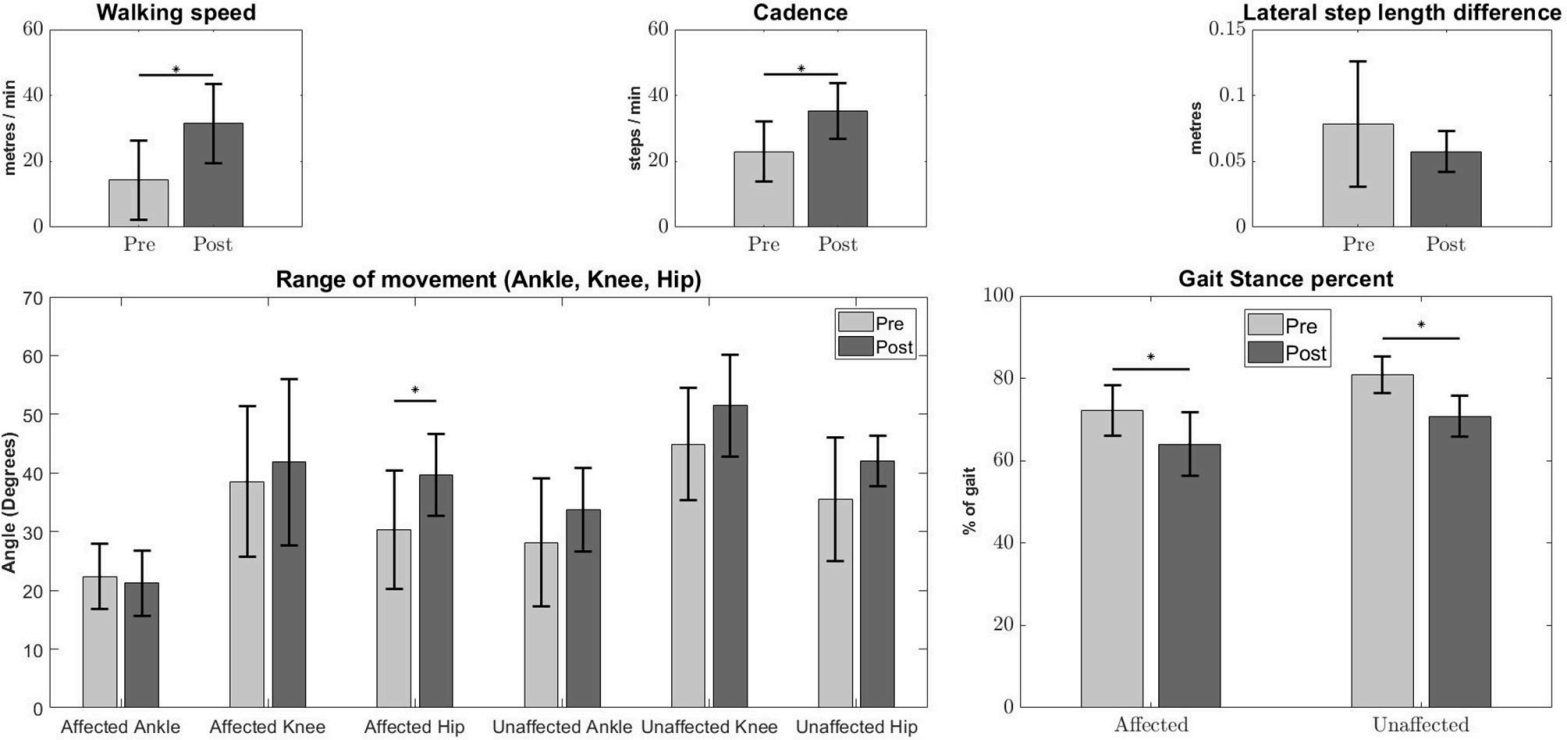
Kinematic measures Pre-Post conditions. Error bars denote the standard deviation. Improvements were observed in walking speed **(Top Left)**, step cadence **(Top Middle)**, stance duration percentage of affected and unaffected side **(Bottom Right)**, and Pre-Post affected hip angles **(Bottom Left)**. The Lateral step length difference **(Top Right)** and other joint angles **(Bottom Left)** do not show any significant differences. The asterisks indicate statistical significance.

Significant improvement in the kinematic measurements of the patients was observed (Figure 1). Statistically significant increase was observed in walking speed [Pre:14.36 ± 12, Post:31.47 ± 12.11m/min, (*p* < 0.05)] and step cadence [Pre:22.95 ± 9.04, Post:35.32 ± 8.45steps/min, (*p* < 0.05)], together with a decrease in the percentage of stance duration, in relation to the whole gait cycle [Pre Affected:72.17 ± 6.12, Post Affected:64.04 ± 4.51%, (*p* < 0.05)] [Pre Unaffected:80.89 ± 7.63, Post Unaffected:70.76 ± 4.96%, (*p* < 0.05)]. There was no significant improvement in absolute lateral difference of step length. [Pre:0.0783 ± 0.0473, Post:0.0575 ± 0.0153m, (*p* > 0.05)]. Only the range of movement for the affected hip show significant improvement [Pre Affected Hip:30.33 ± 10.15, Post Affected Hip:39.63 ± 6.98 degrees(*p* < 0.05)].

The FIM-Locomotion score, FIM-Motor (General) score, FMA-LE scores improved after robotic intervention (Table 2). Only 1 patient (S7) did not show an improvement in FIM-Locomotion scores, however, the other measures (FIM-Motor(General) and FMA-LE) indicated improvement.

**Table 2:** Clinical Measures Pre-Post robotic intervention.

	**FIM- Locomotion (Pre)**	**FIM- Locomotion (Post)**	**FIM-Motor (General) (Pre)**	**FIM-Motor (General) (Post)**	**FMA-LE (Pre)**	**FMA-LE (Post)**
S1	1	**3**	46	**73**	13	**18**
S2	1	**5**	40	**82**	19	**26**
S3	1	**2**	40	**55**	18	**28**
S4	2	**7**	52	**77**	26	**29**
S5	2	**7**	78	**90**	20	**27**
S6	1	**6**	66	**83**	21	**25**
S7	1	1	53	**62**	14	**22**
S8	1	**5**	50	**65**	17	**20**

*Numbers in bold denote improvement in scores*.

### 3.2. Number of muscle synergies in patients

**Figure 2:**
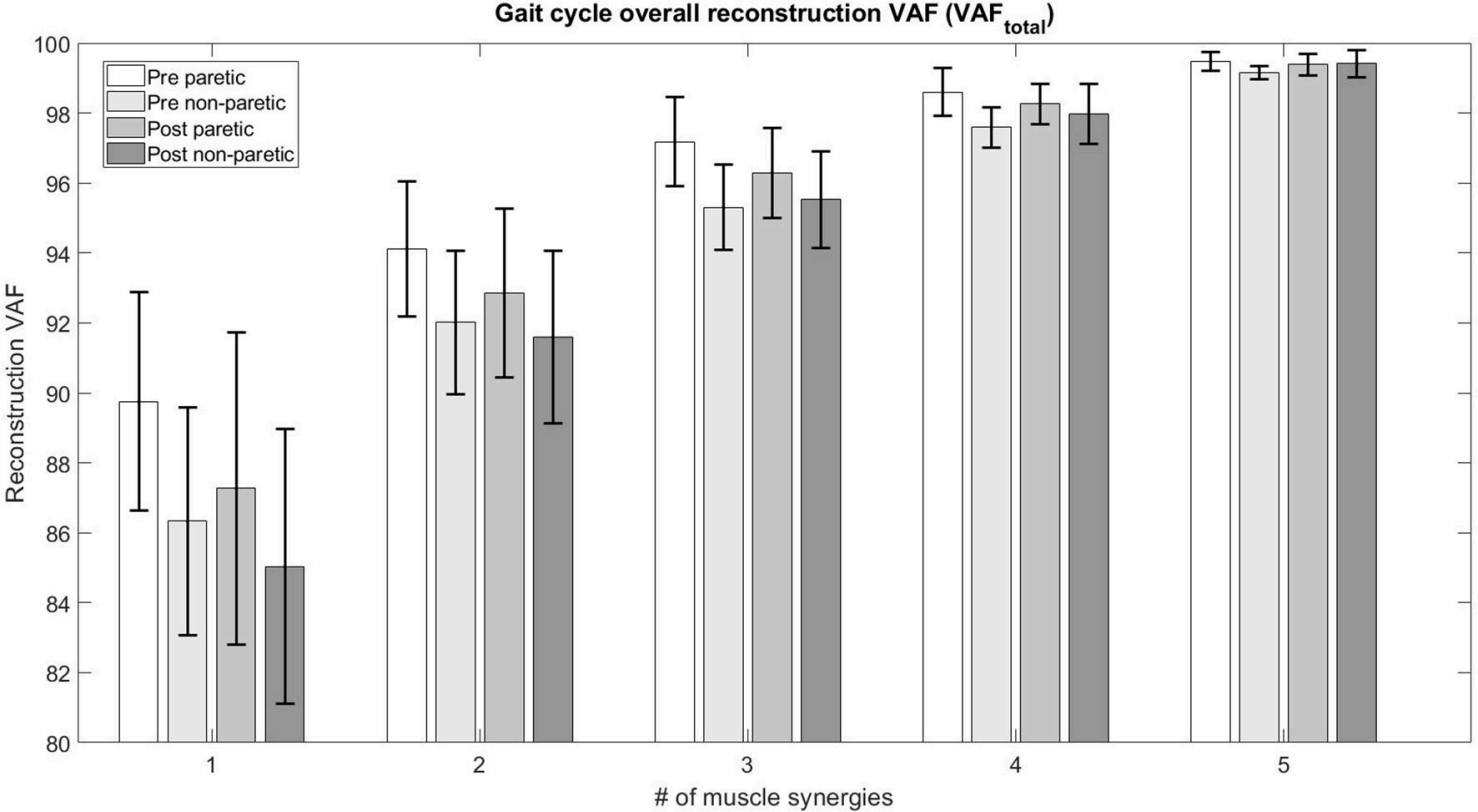
Total variability accounted for (VAF_*total*_) based on the number of muscle synergies.

**Figure 3:**
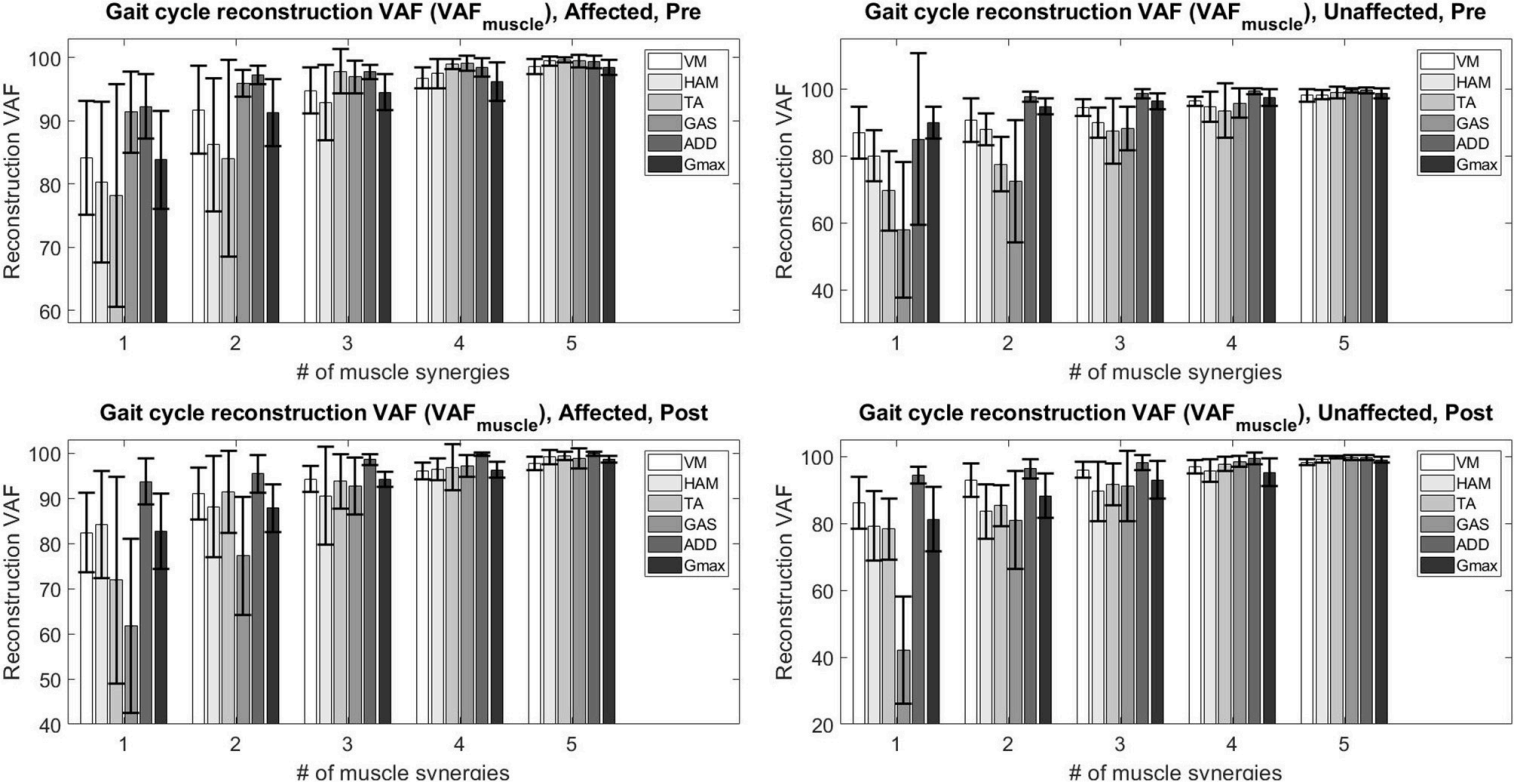
Total variability accounted for (VAF_*muscle*_) based on the number of muscle synergies and condition.

The figure below (Figure 2) details the mean overall reconstruction VAF and mean reconstruction VAF for each muscle vector (Figure 3) according to the number of muscle synergies for all subjects. Error bars denote the standard deviation.

The table below (Table 3) details the change in the number of muscle synergies per subject in different conditions. The number of synergies selected for each subject is based on both the overall reconstruction VAF (VAF_*total*_ > 90%) and local reconstruction VAF (VAF_*muscle*_ > 75%) (Torres-Oviedo and Ting, 2007) for each subject. We found that 3 subjects required an increase of the number of muscle synergies by 1, No change for 3 subjects, and 1 subject required a decrease in the number of muscle synergies for their affected side. The exception to this case is S3, reduced the number of muscle synergies by 1 after therapy. We also found changes in the unaffected side of patients, with S2, S3, and S4 showing a decrease in muscle synergies. The exception to this case were S5 and S6, who had an increase in the number of muscle synergies in the unaffected side.

**Table 3:** Number of muscle synergies required pre and post robotic intervention for affected and unaffected sides.

	**Affected Pre**	**Post**	**Change**	**Unaffected Pre**	**Post**	**Change**
S1	1	**2**	+1	2	2	0
S2	2	2	0	3	**2**	−1
S3	3	**2**	−1	4	**2**	−2
S4	2	**3**	+1	3	**2**	−1
S5	3	**4**	+1	2	**4**	+2
S6	3	3	0	3	**4**	+1
S7	1	1	0	2	2	0
S8	1	**3**	+2	3	3	0

*Bold numbers indicate the change in the number of muscle synergies*.

To determine the number of synergies for comparison between sides per subject, we selected the maximum number of synergy for both the affected and unaffected side in the comparison condition (e.g., In Table 3, the number of synergy for S2 is determined to be 3 for Side Similarity in the pre-intervention condition, and 2 for the post-intervention condition. However, for the same subject, the number of synergies for Pre-Post Similarity comparisons would be 3 for the unaffected side).

### 3.3. Muscle synergies, EMG waveforms and kinematics

**Figure 4:**
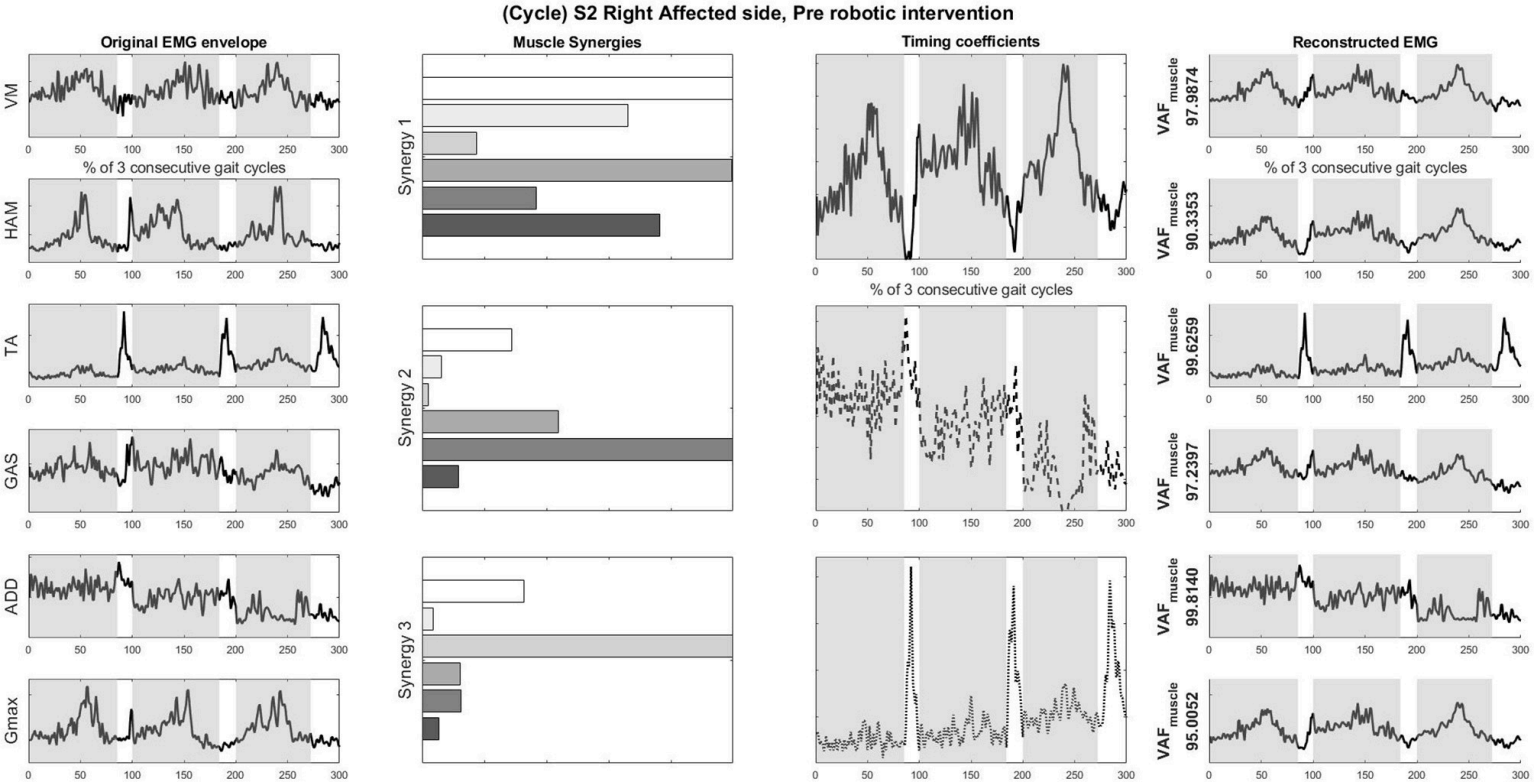
Original EMG envelope, muscle synergies, timing coefficients and Reconstructed EMGs for affected side, pre HAL intervention, computed by NNMF with 3 synergies. Each bar of the synergy set is matched with the order of muscles in the “Original EMG” (Left) column, namely (from top bar to bottom bar) VM, HAM, TA, GAS, ADD, Gmax. Similarly, each plot in the “Individual Reconstructed EMG” column is matched with the order of the muscles in the “Original EMG” (Left) column. Each line pattern represents the reconstructed EMG from each motor module. Shaded areas denote stance phases.

**Figure 5:**
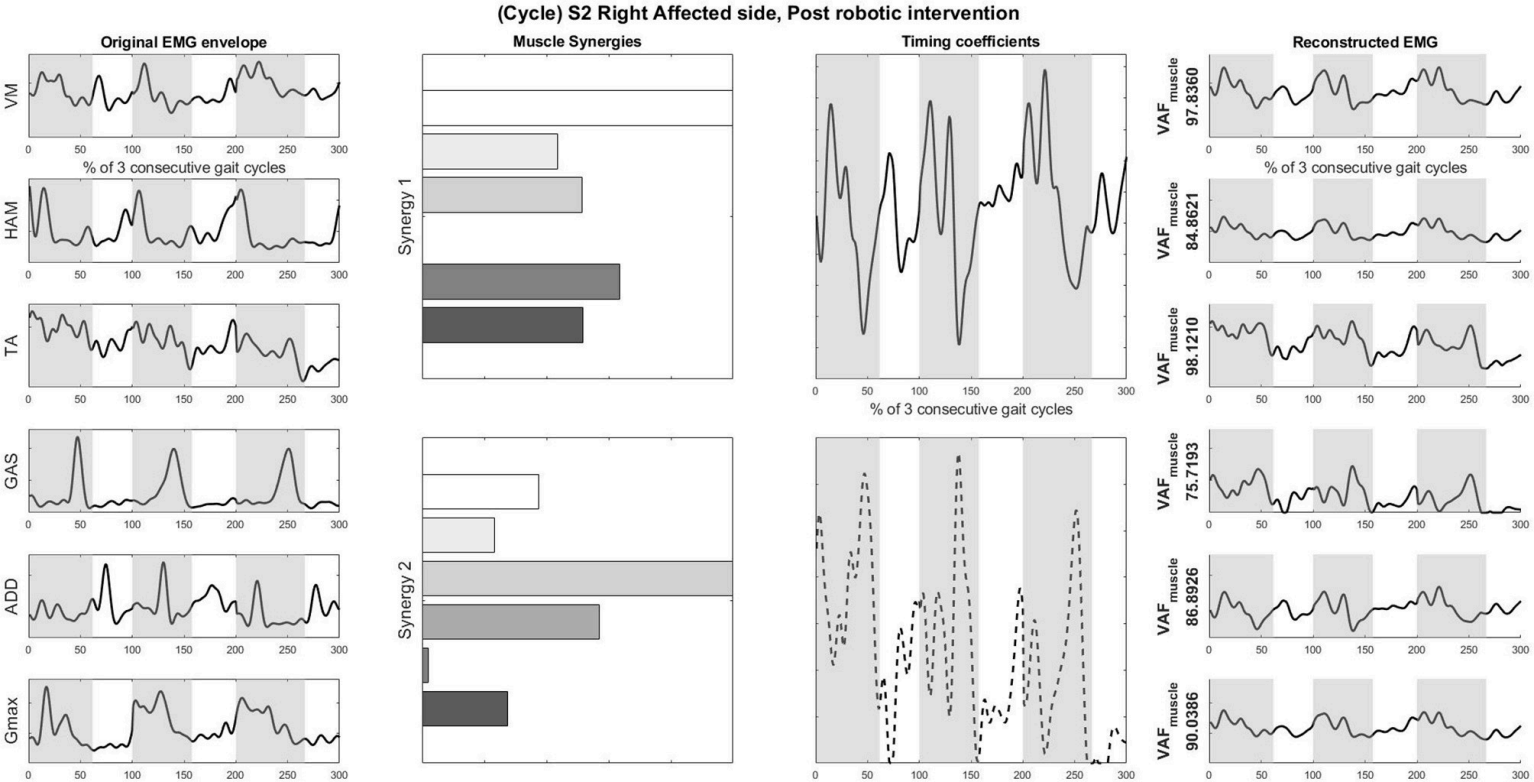
Original EMG envelope, muscle synergies, timing coefficients and Reconstructed EMGs for affected side, post HAL intervention, computed by NNMF with 2 synergies. Each bar of the synergy set is matched with the order of muscles in the “Original EMG” (Left) column, namely (from top bar to bottom bar) VM, HAM, TA, GAS, ADD, Gmax. Similarly, each plot in the “Individual Reconstructed EMG” column is matched with the order of the muscles in the “Original EMG” (Left) column. Each line pattern represents the reconstructed EMG from each motor module. Shaded areas denote stance phases.

**Figure 6:**
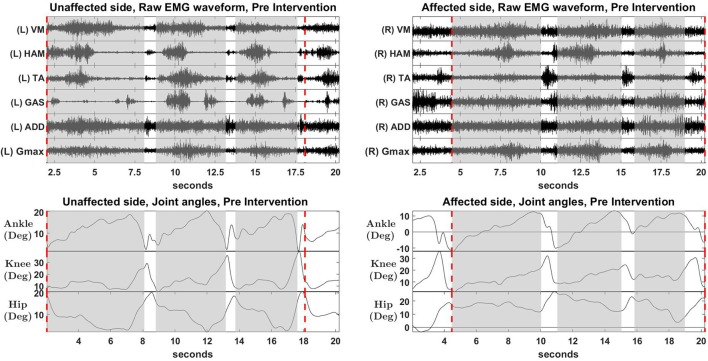
Raw EMG waveform and joint angles, pre intervention. Positive values in angles indicate flexion, while negative values indicate extension.

**Figure 7:**
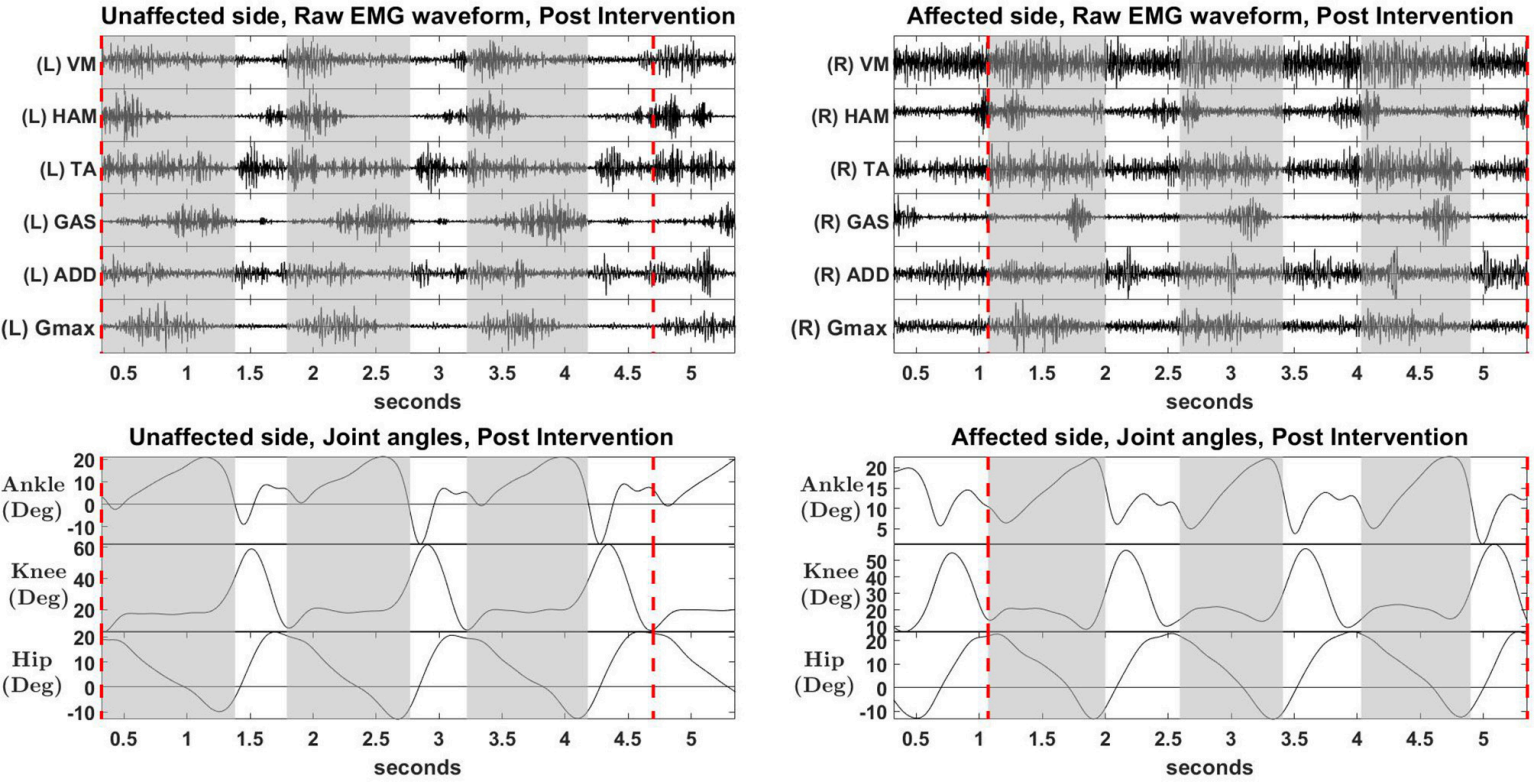
Raw EMG waveform and joint angles, post intervention. Positive values in angles indicate joint flexion, while negative values indicate extension.

S2 was picked as the representative subject for reporting as this subject displays the greatest improvement in FIM (General) scores and reasonably good improvement in both FIM locomotion and FMA lower limb scores. Figures 4, 5 shows the muscle synergy vectors, timing coefficients, original EMG envelopes and reconstructed EMGs, for the affected side of S2, during the pre-intervention and post-intervention conditions respectively. Raw EMG waveforms and joint angles for S2 in the pre-intervention and post-intervention conditions (Figures 6, 7, respectively) were presented.

Overall reconstruction VAF for S2 is high (>90%) for both conditions, although certain abnormal looking EMG envelopes can be seen. To explain them, the raw EMG waveforms, and their corresponding joint angles, for both pre and post intervention conditions, are examined (Figures 6, 7). Due to ethical reasons, we are unable to provide the raw EMG values, hence the vertical axes of EMG plots remain unlabeled. In the Pre-intervention condition (Figure 6, Affected side), subject's VM and GAS muscles are active for most of the gait cycle (**Stance percent**: 80.56 ± 7.24). This can be observed in the slow rate of increase in the knee angles. ADD muscles are also highly active in the pre-intervention condition, so as to provide stability for the hip joint, where hip angles hover around values between 10 and 20° for most of the stance phase. In the post-intervention condition (Figure 7, Affected Side), the subject has an overly active TA post rehabilitation, contributing to abnormal dorsal flexion of the ankle throughout the gait cycle. We observed the VM muscles are active longer than usual in the stance phase. The timing of the GAS muscles were later, as compared to the unaffected side. The HAM muscles appear to be weaker than the unaffected side, where short activations were noted. The knee angle trajectory shows a slight parabolic curve on the Affected knee, as opposed to a relatively straight trajectory on the Unaffected knee.

### 3.4. Synergy changes after robotic intervention

Muscle synergy similarity was quantified with the Pearson correlation coefficient (*r*) to provide an overall view of the lateral symmetry of the muscle synergies.

**Figure 8:**
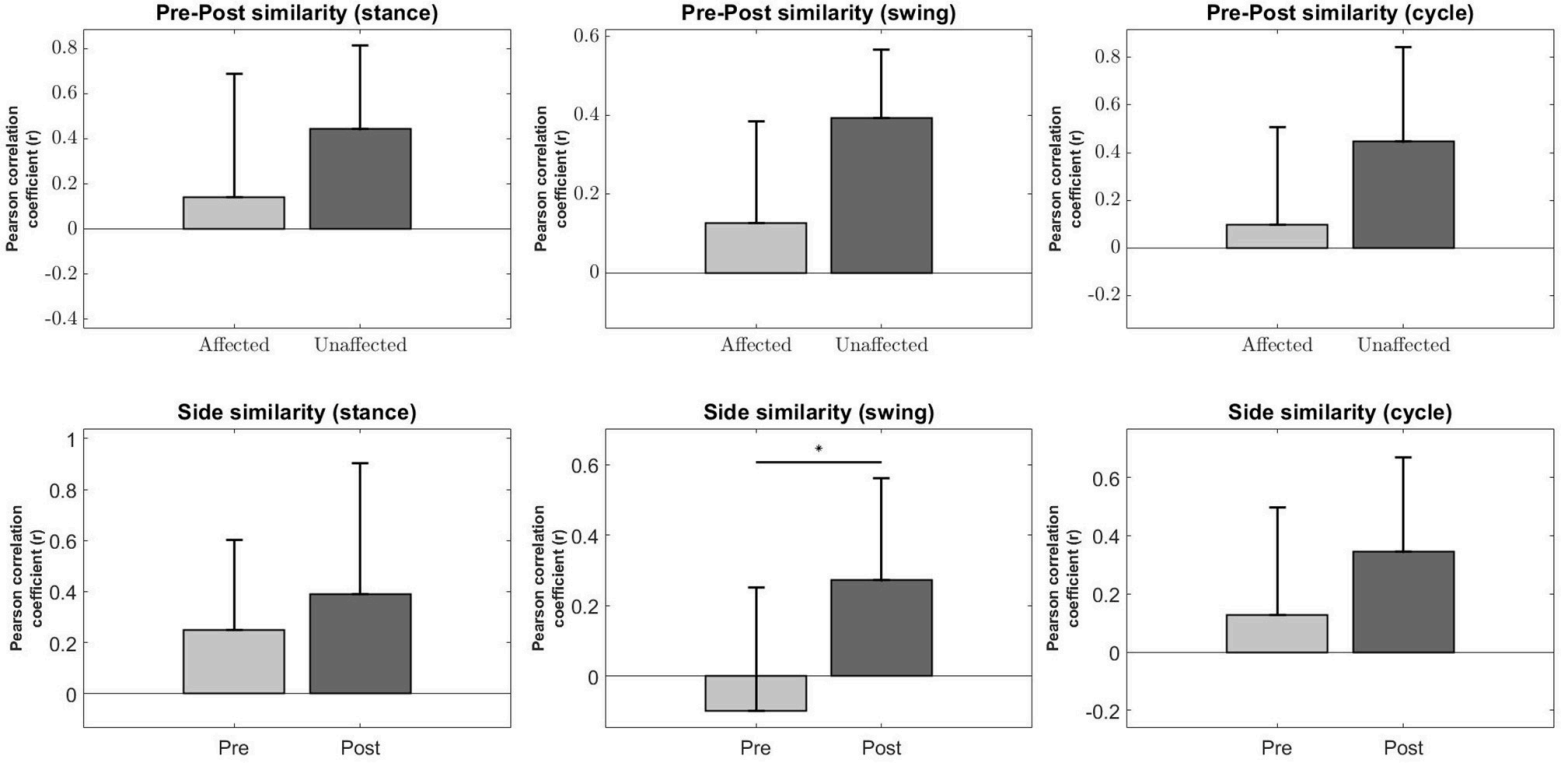
Synergy comparison between conditions. Error bars denote standard deviation. Pre-Post comparison of *r* between affected and unaffected side during Stance, Swing and Full gait cycle **(Top Row)**. Side comparison of *r* before and after robotic intervention during Stance, Swing and Fully gait cycle **(Bottom Row)**. The asterisks indicate statistical significance.

A significant increase in the bilateral symmetry in the swing phase was observed (Pre : −0.0987 ± 0.349, Post : 0.272 ± 0.291, *p* < 0.05) (Figure 8, Second column, Bottom). However, no significance were found for other phases of gait, although a upward trend can be observed. **Stance** (Pre : 0.251 ± 0.352, Post : 0.39 ± 0.514, *p* > 0.05), **Cycle** (Pre : 0.129 ± 0.368, Post : 0.344 ± 0.323, *p* > 0.05).

Pre-Post similarities between the affected and unaffected side for all phases were also not significant, although the variability in *r* for the affected side is much higher than the unaffected side, possibly indicating a greater change in muscle synergies after robotic intervention. **Stance** (Pre : 0.142 ± 0.548, Post : 0.443 ± 0.371, *p* > 0.05), **Swing** (Pre : 0.128 ± 0.257, Post : 0.393 ± 0.174, *p* > 0.05), **Cycle** (Pre : 0.0988 ± 0.41, Post : 0.446 ± 0.397, *p* > 0.05) (Figure 8, Top Row).

**Figure 9:**
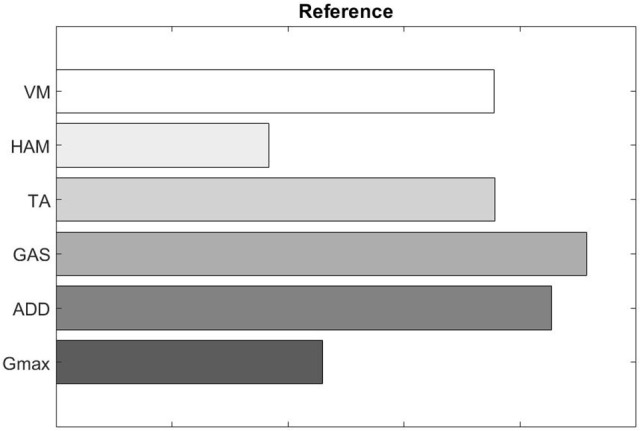
Reference plot displaying the arrangement of muscles in Figures 10, 11.

**Figure 10:**
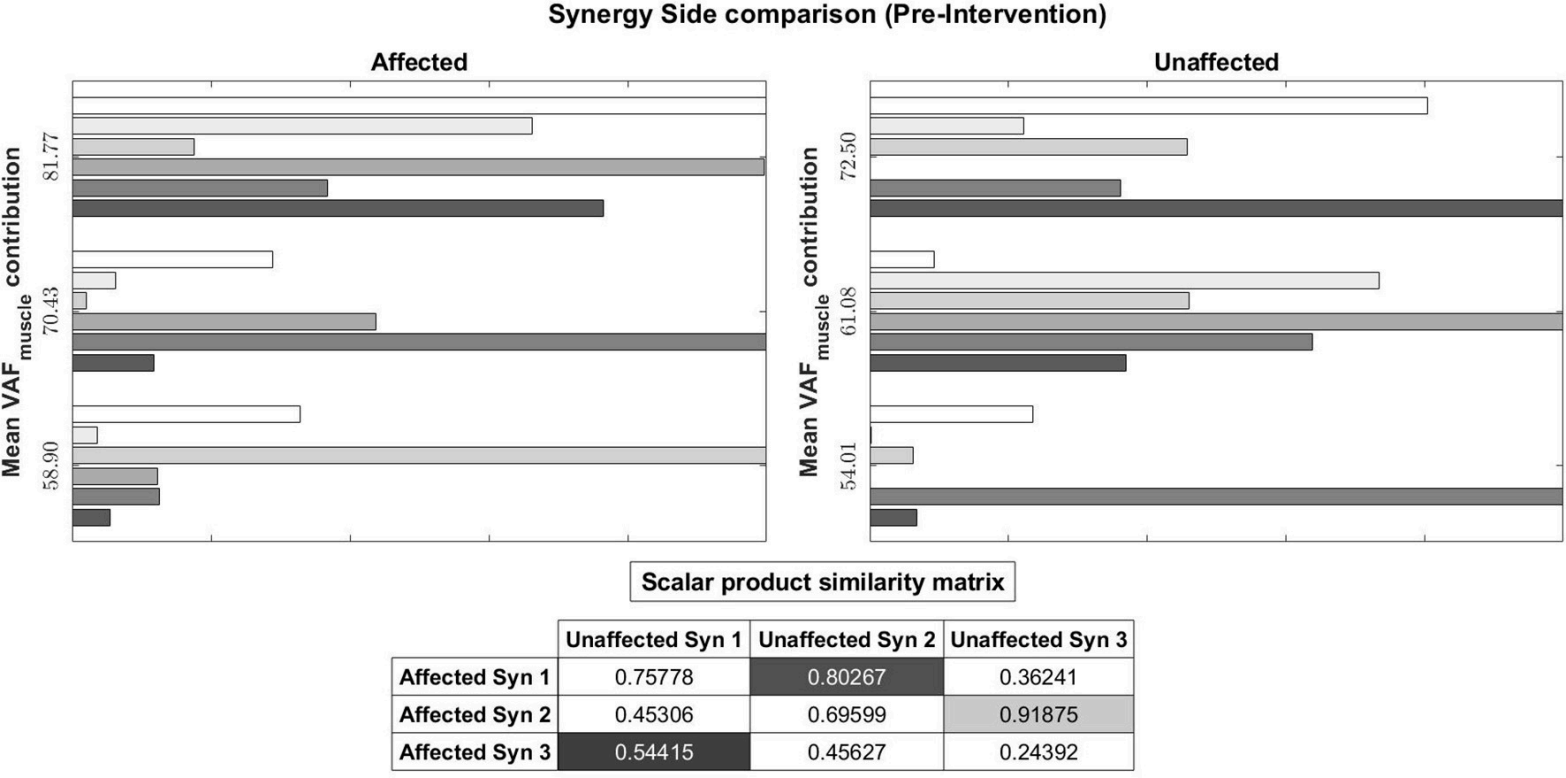
Muscle synergy vectors of Subject 2, Pre intervention, Full Gait cycle. VAF to original states the contribution of each synergy to the original EMG. A higher value indicates a higher contribution. The scalar product similarity matrix shows results of comparing each synergy vector on the Affected side with all synergy vectors on the Unaffected side. Muscle synergies with the highest score are selected, matched and removed from the pool of muscle synergies. This process continues until no more muscle synergies are left to match. The synergies are matched in the sequence given by the color codes: 1st, Light gray; 2nd, Dark gray; 3rd, Black. The 1st synergy on the affected side is matched with the 2nd synergy on the unaffected side. Similarly, the 3rd synergy on the affected side is matched with the 1st synergy on the unaffected.

**Figure 11:**
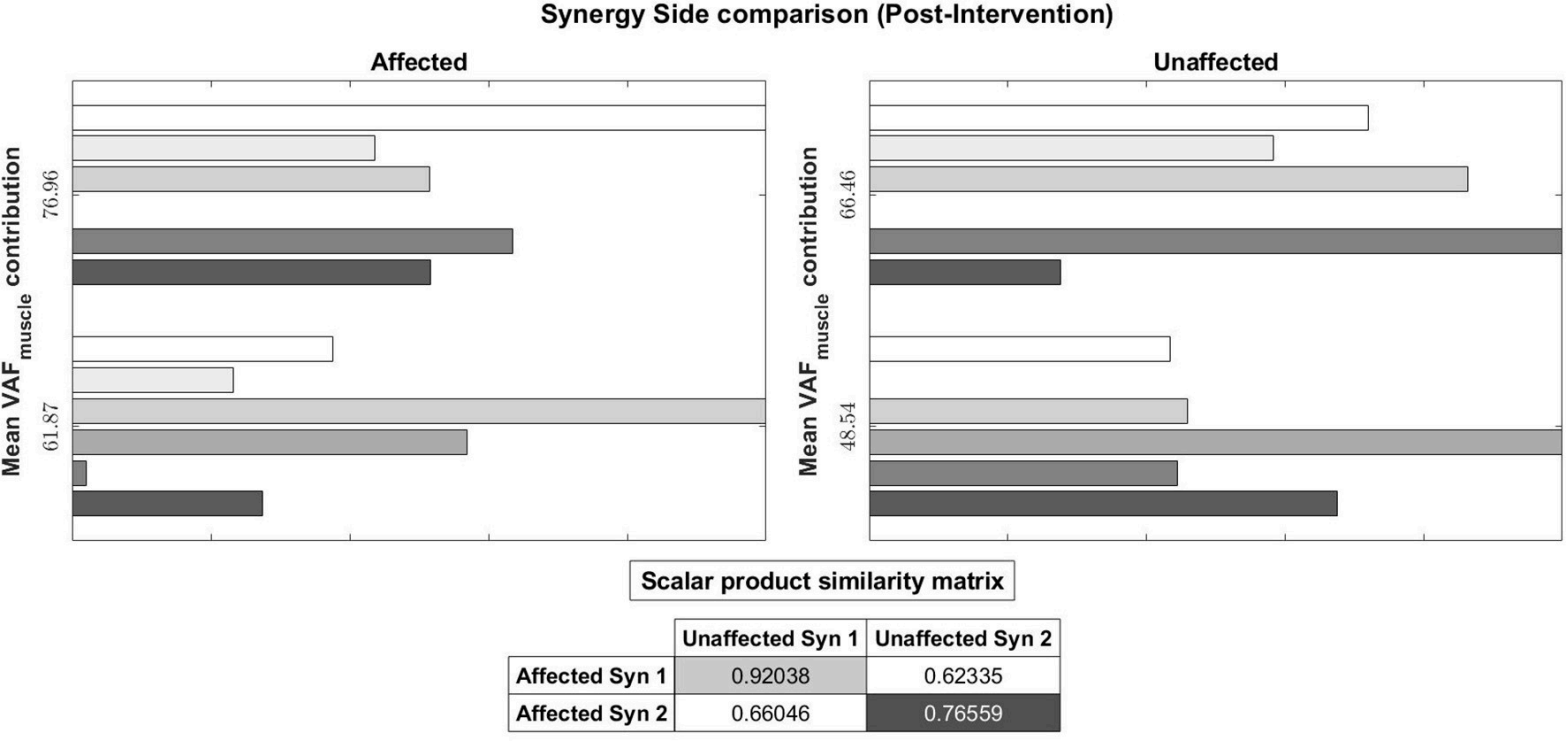
Muscle synergy vectors of Subject 2, Post intervention, Full Gait cycle. With the same muscle synergy matching method, The order of muscle synergies for Subject 2 on the affected side was matched with the order of muscle synergies on the unaffected side (1st synergy on the affected side is matched with the 1st synergy on the unaffected and so on).

We also evaluated muscle synergy vectors on an individual basis as well. **Figures 10**, **11** show our results for Subject 2. Figure 9 labels the muscles shown in Figures 10, 11.

Pre-intervention (Figure 10), it can be observed that muscle synergies on the affected side are different from muscle synergies on the unaffected side, when sorted by task contribution levels. For example, the first synergy on the affected side has that largest similarity to the second synergy on the unaffected side. This differences in task contribution levels of similar muscle synergies could lead to the observed asymmetric gait (Figure 6).

In the post-intervention condition (Figure 11), similar muscle synergies, denoted by a high scalar dot product value, now has similar task contribution levels, and this probably lead to a more symmetric gait (Figure 7).

## 4. Discussions and conclusions

Our current study attempts to provide a method to measure lateral symmetry by evaluating the number of synergies for each side of the body and contents of the muscle synergy vectors. We think this is a better representation of how the patient is moving, since these synergies quantify muscle activations. By comparing these muscle synergies, we can assess gait symmetries of patients. Although the usual method for previous studies to assess the performance of stroke patients is to compare them with healthy subjects (Bowden et al., 2010; Gizzi et al., 2011; Routson et al., 2013; Hayes et al., 2014; Barroso et al., 2016; Pérez-Nombela et al., 2017), we show that lateral symmetry can still be used as a relative measure by comparing the sides of the patients. Future considerations would be to analyze healthy subjects to test the accuracy of this method.

The increase in lateral symmetry is also associated with the improvement in clinical assessment and gait characteristics. This shows that robotic intervention is helpful for stroke patients (Figure 1 and Table 2). Among the kinematic measures, only absolute lateral difference of step length did not show significant improvement (Figure 1, Top Right). This is because we think the step length variable, which is the basis for absolute step length difference, is too vague as a measure. As was described in section 2.5, this variable does not consider the side favored by the patient during walking.

In our study, we utilized a sorting method to arrange muscle synergies according to their contributions for a particular task. One reason for the sorting is because the NNMF algorithm randomly orders the factorized synergies. Another reason is to account for the lack of age-matched controls. Although cluster analysis (Martino et al., 2015; Cappellini et al., 2016) is also an important method to match muscle synergies and indicate the presence of similar muscle synergies on both side of the body, muscle synergies should also be evaluated in context of the task. Nazifi et al. (2017) and Torres-Oviedo and Ting (2010) show that task-specific muscle synergies are dynamically recruited for different tasks, in addition to common muscle synergies found in all subjects. Chvatal and Ting (2013) noted that muscle synergies may be recruited by different neural circuits for a common motor task. These studies suggest that the task might influence the recruitment of muscle synergies, hence analysis should be done in context of the task. We do not expect our subjects to possess similar muscle synergies, due to differences in descending neural commands from the motor cortex caused by stroke. Hence, sorting muscle synergies would help standardize comparison to the task level, allowing muscle synergies with similar contribution levels on both sides of the body to be compared.

Our results show that stroke patients have reduced number of muscle synergies on their affected side as compared to their unaffected side pre intervention (Table 3, Affected Pre and Unaffected Pre). This agrees with the previous studies on MSA of stroke patients (Clark et al., 2010; Cheung et al., 2012). However, we have also observed patients who have decreased number of synergies on their unaffected side pre intervention [Table 3, S3 (Affected Side), and S2, S3, S4 (Unaffected Side)]. Although this appears to be contradictory, a study by Hashiguchi et al. (2016) found that subacute stroke patients exhibit both fractionation and merging of muscle synergies. They conclude that the number of muscle synergies do not consistently change with the recovery phase. They also found that the merging of synergies is associated with decrease in muscle strength and range of movement in the ankle joint, while fractionation is only related to improvement in the Barthel index.

We also noticed that the unaffected side tends to match the number of synergies on the affected side, with S2, S3, and S4 showing a decrease in the number of synergies, and S5 and S6 showing an increase in the number of synergies. This seems to agree with our results of patients having increased lateral symmetry in muscle synergies while walking. We think that the decrease in the number of synergies on the unaffected side could be due to the central nervous system trying to match the number of synergies on both sides of the body (Decrease in unaffected side of S2, S3, S4). Similarly, when the affected side sees an increase in the number of synergies, the unaffected side would probably require an increase in the number of synergies as well, in order to cater for the increased variety of movement (Increase in unaffected side of S5 and S6). A possible explanation for this phenomena is put forth by Graziadio et al. (2012), who studied bilateral reorganization of the corticospinal system of stroke patients with hemiparesis. They found that the corticospinal system appears to prioritize symmetrical recovery, even if it is achieved at the expense of the non-lesioned side.

We think that once the affected side regains sufficient motor function, there is no need for the unaffected side to compensate for the affected side, hence leading to a change in the number and contents of synergies, thus, achieving gait symmetry. This could be beneficial for the patients, as gait asymmetries would lead to further complications in future if left untreated (Verma et al., 2012). We hypothesize that this might be the central nervous system's way of regaining symmetry. Indeed, results by Clark et al. (2010) suggest that the organization of muscle synergies are similar in the legs of both healthy and post-stroke patients, with the only difference being the ability to activate muscle synergies independently, where reduction in this ability leads to merged synergies. This is also seen in the study of Cheung et al. (2012). However, associating the number of degrees of freedom to number of muscle synergies seem to contradict our results, where we observed a reduction in the number of synergies post robotic intervention, and those of Hashiguchi et al. (2016), which indicate fractionation can also occur in postacute stroke patients. As Cheung et al. (2012) pointed out, the motor system is a complex mix of descending and ascending neural pathways that interact with each other, and that changes also occur in subcortical areas. More work in understanding muscle synergies in the context of both the cortical and subcortical neural circuits have to be done before any concrete conclusions can be drawn.

Our hypothesis of HAL was that, by its function of actually performing intended motion in real time based on the detected peripheral neuromuscular activity, it can assist neurorehabilitation of the original neuro-muscular motor function of the affected limb. This is in contrast to conventional physical therapy, in which the unaffected side was trained to perform compensatory motions, with orthoses and/or walking aids prescribed to help regain functional independence in daily life (Verma et al., 2012). It was also noted by Verma et al. (2012) that the adult human brain is capable of reorganization after stroke and can be manipulated with movement stimuli involving lower limbs. Shimizu et al. (2017) showed that recovery of neuromuscular activity is possible even in patients with chronic complete spinal cord injury with quadri/paraplegia. They used HAL to allow patients to trigger voluntary ambulation with residual muscle activations in their arms. This supports our hypothesis of HAL's effect on neurorehabilitation after stroke observed in this study.

It is also widely discussed that the synergy modules of muscular activation extracted by NNMF represents the way the central nervous system organizes the coordinated control of multiple muscles by descending commands to the peripheral (Ting et al., 2015). The improvement of lateral synergy after robotic intervention using HAL shown in this study suggests possible contribution of HAL in the improvement of neuronal organization of gait by the central nervous system, in the acute phase post-stroke patients.

### 4.1. Limitations of study

Limitation of the study includes the lack of control patients who did not receive HAL treatment. However, we do note that Louie and Eng (2016) have performed an extensive review of various clinical trials utilizing robotic intervention for post-stroke treatment, with findings that robotic intervention is safe and beneficial for stroke patients. Hence, this study is focused on developing methods to quantify the effects of robotic intervention. Nevertheless, comparison to control group and investigation of synergy organization during sessions remain for future consideration.

We acknowledge that the variety of impaired gait in stroke patients cannot be fully captured with 8 subjects. However, our study would like to show that muscle synergies are able to quantify gait asymmetries in stroke patients and hope that this method would inspire others to use and refine our methods. That said, increasing the number of subjects remains a consideration for future studies.

## Author contributions

CT and HK collected, analyzed and interpreted the data; wrote and drafted the manuscript. HW and AM planned and administered HAL treatment. AM diagnosed the patients and prescribed HAL treatment. MY provided important comments for the clinical part of the study and helped developing HAL treatment. YS originally developed the robot suit HAL and conceived the idea of HAL treatment. KS designed the analysis and provided essential insight for the paper. All authors made critical revisions of the manuscript and approved the final version.

### Conflict of interest statement

YS is the C.E.O., shareholder, and director of CYBERDYNE Inc. which produces the robot suit HAL. CYBERDYNE was not involved in study design, data collection, analysis, writing or submission of this article. The other authors declare that the research was conducted in the absence of any commercial or financial relationships that could be construed as a potential conflict of interest.
