# Corrigendum: Lateral Symmetry of Synergies in Lower Limb Muscles of Acute Post-stroke Patients After Robotic Intervention

**DOI:** 10.3389/fnins.2020.00113

**Published:** 2020-02-21

**Authors:** Chun Kwang Tan, Hideki Kadone, Hiroki Watanabe, Aiki Marushima, Masashi Yamazaki, Yoshiyuki Sankai, Kenji Suzuki

**Affiliations:** ^1^Artificial Intelligence Laboratory, University of Tsukuba, Tsukuba, Japan; ^2^Center for Innovative Medicine and Engineering, University of Tsukuba Hospital, Tsukuba, Japan; ^3^Department of Neurosurgery, Faculty of Medicine, University of Tsukuba, Tsukuba, Japan; ^4^Department of Orthopaedic Surgery, Faculty of Medicine, University of Tsukuba, Tsukuba, Japan; ^5^Center for Cybernics Research, University of Tsukuba, Tsukuba, Japan; ^6^Faculty of Engineering, University of Tsukuba, Tsukuba, Japan

**Keywords:** muscle synergies, gait symmetry, stroke rehabilitation, robotic intervention, hybrid assistive limb

In the original article, there was a mistake in Table 1 as published. **The gender of S7 is wrongly listed as “M,” where it should be “F.”** The corrected Table 1 appears below.

**Table 1:** Participants characteristics.

**ID**	**Age (years)**	**Gender**	**Diagnosis**	**Affected side**	**FAC before HAL**	**Onset-HAL duration (days)**
S1	67	F	ACI	L	1	10
S2	52	F	SH	R	2	17
S3	71	F	BSI	L	1	11
S4	55	M	LI	L	2	10
S5	55	F	ABSI	L	3	16
S6	43	M	LI	R	2	11
S7	51	F	ACI	R	2	18
S8	80	M	ACI	R	2	16

*Diagnosis was Atherothrombotic Cerebral Infarction (ACI), Subcortical Hemorrhage (SH), Brain Stem Infarction (BSI), Lacunar Infarct (LI) or Atherothrombotic Brain Stem Infarction (ABSI)*.

Additionally, there was a mistake in Table 2 as published. **The FMA-LE (Pre) of S6 is wrongly listed as “23,” where it should be “21.”** The corrected Table 2 appears below.

**Table 2:** Clinical Measures Pre-Post robotic intervention.

	**FIM-Locomotion (Pre)**	**FIM-Locomotion (Post)**	**FIM-Motor (General) (Pre)**	**FIM-Motor (General) (Post)**	**FMA-LE (Pre)**	**FMA-LE (Post)**
S1	1	**3**	46	**73**	13	**18**
S2	1	**5**	40	**82**	19	**26**
S3	1	**2**	40	**55**	18	**28**
S4	2	**7**	52	**77**	26	**29**
S5	2	**7**	78	**90**	20	**27**
S6	1	**6**	66	**83**	21	**25**
S7	1	1	53	**62**	14	**22**
S8	1	**5**	50	**65**	17	**20**

*Numbers in bold denote improvement in scores*.

The authors apologize for this error and state that this does not change the scientific conclusions of the article in any way. The original article has been updated.

